# Hexacarbonyl-2κ^3^ *C*,3κ^3^ *C*-di-μ_3_-sulfido-tetra­kis­(tetra­hydro­furan-1κ*O*)calcium­diiron(II)(*Fe*—*Fe*)

**DOI:** 10.1107/S1600536812048039

**Published:** 2012-11-30

**Authors:** Mikhail A. Ogienko, Dmitry Yu. Naumov, Sergey N. Konchenko

**Affiliations:** a Nikolaev Institute of Inorganic Chemistry, SB Russian Academy of Sciences, Akad. Lavrentiev prospekt 3, Novosibirsk 90, 630090 Russian Federation; b Novosibirsk State University, Pirogov st. 2, Novosibirsk 90, 630090 Russian Federation

## Abstract

Reaction between [Fe_2_(μ-S_2_)(CO)_6_] and [Ca(thf)_4_(dpp-BIAN)] [dpp-BIAN = 1,2-bis-(2,6-diisopropyl­phenyl­imino)­acenaphthene and thf = tetra­hydro­furan] proceeds as a redox process *via* a two-electron reduction of [Fe_2_(μ-S_2_)(CO)_6_] and a two-electron oxidation of (dpp-BIAN)^2−^, resulting in the formation of the title heterometallic trinuclear cluster, [CaFe_2_(μ_3_-S)_2_(C_4_H_8_O)_4_(CO)_6_], and neutral dpp-BIAN. In the cluster, the Ca^II^ atom is connected to two S atoms of an Fe_2_S_2_ core [Ca—S = 2.7463 (8) and 2.7523 (8) Å]. No Fe—Ca bonds are formed [Fe⋯Ca = 3.6708 (6) and 3.5802 (6) Å]. There are five close C–H⋯O–C contacts in the crystal structure.

## Related literature

For the synthesis and structure of [Fe_2_(CO)_6_(μ-S_2_)], see: Hieber & Beck (1958 ▶); Seyferth *et al.* (1982 ▶), and of [Ca(thf)_4_(dpp-BIAN)], see: Fedushkin *et al.* (2003 ▶). For the synthesis and structures of related heterometallic clusters with an Fe_2_S_2_ core, see: Konchenko *et al.* (2010 ▶); Cowie *et al.* (1989 ▶); Veith *et al.* (2005 ▶); Eremenko *et al.* (1994 ▶); Pasynskii *et al.* (1993 ▶). For FeS-clusters as model compounds for active sites of hydrogenases, see: Gloaguen & Rauchfuss (2009 ▶).
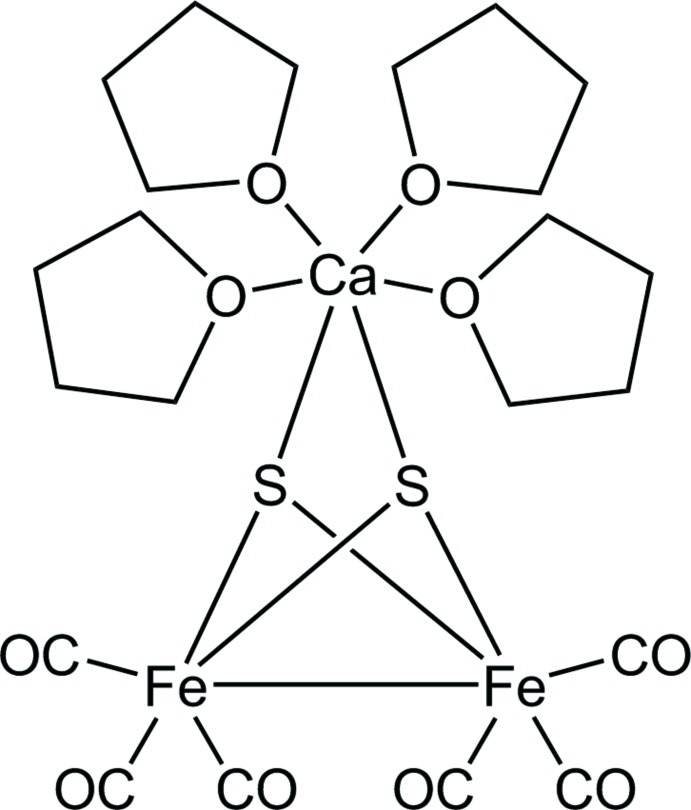


## Experimental

### 

#### Crystal data

[CaFe_2_S_2_(C_4_H_8_O)_4_(CO)_6_]
*M* *_r_* = 672.38
Orthorhombic, 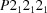

*a* = 10.9189 (4) Å
*b* = 12.4167 (5) Å
*c* = 21.4545 (9) Å
*V* = 2908.7 (2) Å^3^
*Z* = 4
Mo *K*α radiation
μ = 1.37 mm^−1^
*T* = 150 K
0.25 × 0.11 × 0.08 mm

#### Data collection

Bruker–Nonius X8 APEX CCD area-detector diffractometer
Absorption correction: multi-scan (*SADABS*; Bruker, 2004 ▶) *T*_min_ = 0.726, *T*_max_ = 0.899
21313 measured reflections
5951 independent reflections
5319 reflections with *I* > 2σ(*I*)
*R*_int_ = 0.033

#### Refinement

*R*[*F* ^2^ > 2σ(*F* ^2^)] = 0.027
*wR*(*F* ^2^) = 0.057
*S* = 1.01
5951 reflections
334 parameters
H-atom parameters constrained
Δρ_max_ = 0.36 e Å^−3^
Δρ_min_ = −0.32 e Å^−3^
Absolute structure: Flack (1983) ▶, 2601 Friedel pairs
Flack parameter: 0.006 (11)

### 

Data collection: *APEX2* (Bruker, 2004 ▶); cell refinement: *SAINT* (Bruker, 2004 ▶); data reduction: *SAINT*; program(s) used to solve structure: *SHELXS97* (Sheldrick, 2008 ▶); program(s) used to refine structure: *SHELXL97* (Sheldrick, 2008 ▶); molecular graphics: *DIAMOND* (Brandenburg, 1999 ▶); software used to prepare material for publication: *SHELXTL* (Sheldrick, 2008 ▶).

## Supplementary Material

Crystal structure: contains datablock(s) I, global. DOI: 10.1107/S1600536812048039/zl2517sup1.cif

Structure factors: contains datablock(s) I. DOI: 10.1107/S1600536812048039/zl2517Isup2.hkl

Additional supplementary materials:  crystallographic information; 3D view; checkCIF report

## Figures and Tables

**Table 1:** Selected bond lengths (Å)

Fe1—Fe2	2.5152 (5)
Fe1—S1	2.2999 (7)
Fe1—S2	2.3185 (7)
Fe2—S1	2.3077 (7)
Fe2—S2	2.3110 (7)

**Table 2:** Hydrogen-bond geometry (Å, °)

*D*—H⋯*A*	*D*—H	H⋯*A*	*D*⋯*A*	*D*—H⋯*A*
C34—H34*A*⋯O1^i^	0.99	2.57	3.437 (5)	146
C34—H34*B*⋯O2^ii^	0.99	2.62	3.329 (4)	129
C42—H42*A*⋯O6^iii^	0.99	2.63	3.362 (4)	131
C45—H45*B*⋯O3^iv^	0.99	2.65	3.470 (3)	141
C15—H15*A*⋯O1^v^	0.99	2.67	3.561 (3)	150

Symmetry codes: (i) 
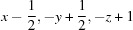
; (ii) 
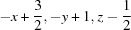
; (iii) 
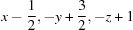
; (iv) 
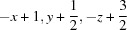
; (v) 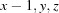.

**Fig. 1.**
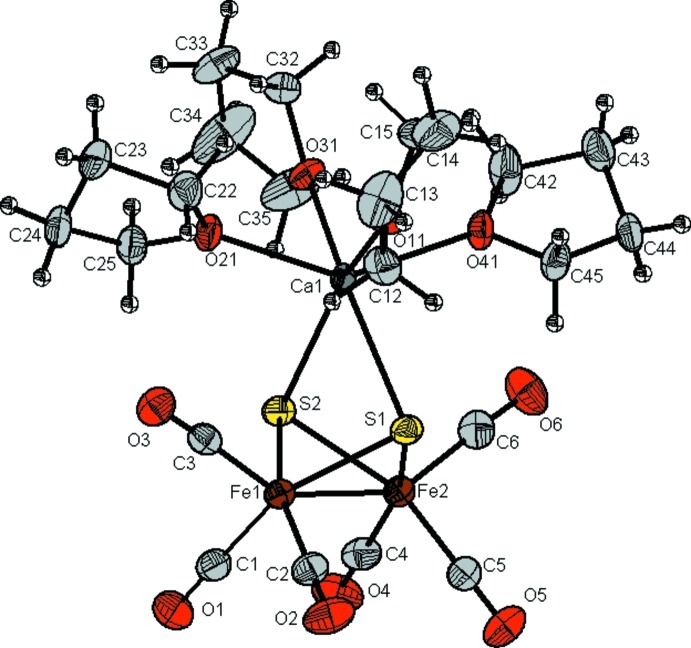
Molecular structure of [Fe2(CO)6(µ3-S)2Ca(thf)4]. Displacement ellipsoids are plotted at the 50% probability level.

**Fig. 2.**
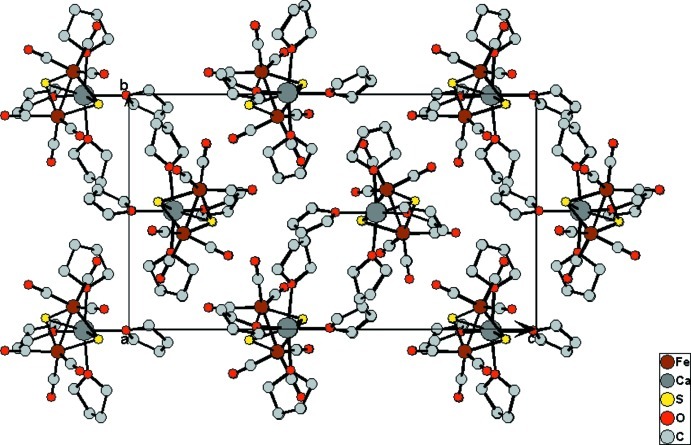
Packing of the structure viewed along a axis. H atoms are omitted for clarity.

## supplementary crystallographic information

### Comment

FeS-clusters are part of the active sites of several enzymes such as hydrogenases. The development of model compounds for these kinds of compounds has attracted the attention of chemists for several decades (Gloaguen & Rauchfuss, 2009). In this context we aimed to study the chemical reduction of [Fe_2_(CO)_6_(µ-S_2_)] with a calcium complex containing the redox active ligand 1,2-bis-(2,6-diisopropylphenylimino)acenaphthene (dpp-BIAN) as the reducing agent. Reduction of [Fe_2_(CO)_6_(µ-S_2_)] with [Ca(thf)_4_(dpp-BIAN)] resulted in the formation of trinuclear calcium iron cluster of the composition [Fe_2_(CO)_6_(µ_3_-S)_2_Ca(thf)_4_] and neutral dpp-BIAN. Formally the process may be considered as a 2-electron reduction of [Fe_2_(CO)_6_(µ-S_2_)] by (dpp-BIAN)^2-^ located in the coordination sphere of Ca^2+^. After 2-electron oxidation (dpp-BIAN)^2-^ becomes neutral dpp-BIAN and is readily substituted by O-donor THF molecules due to the extremely high oxophilicity of Ca^2+^.

The two-electron reduction of [Fe_2_(CO)_6_(µ-S_2_)] by [Ca(thf)_4_(dpp-BIAN)] led to the formation of the heterometallic trinuclear cluster [Fe_2_(CO)_6_(µ_3_-S)_2_Ca(thf)_4_]. In the molecule of the cluster the Ca atom is connected to two S atoms of the Fe_2_S_2_ core (*d*(Ca1–S2) = 2.7463 (8) Å; d(Ca1–S1) = 2.7523 (8) Å). In the case observed here, no Fe–Ca bonds are formed (*d*(Fe2–Ca1) = 3.6708 (6) Å; d(Fe1–Ca1) = 3.5802 (6) Å). All atoms lie in general positions.

The C–O ligands are surrounded by H-atoms from neighboring THF-ligands. There are five close C–H···O–C contacts with O···H distances from 2.571 Å to 2.672 Å (Table 2).

The S–S distances and the S–Fe–Fe–S torsion angle in the Fe_2_S_2_ core depend on the atomic radius and the nature of the heterometal. In case of [Fe_2_(CO)_6_(µ-S_2_)] itself with no heterometal attached the S–S distances average 2.021 (2) Å, the S–Fe–Fe–S torsion angle is 66.89 (2) ° (Eremenko *et al.*, 1994). In the case of [Fe_2_(CO)_6_(µ_3_-S)_2_Pt(PPh_3_)_2_] d(S–S) is 2.86 (4) Å, the S–Fe–Fe–S torsion angle is 97 (2) ° (Pasynskii *et al.*, 1993). For [Fe_2_(CO)_6_(µ_3_-S)_2_Sn(µ-N*t*-Bu)_2_SiMe_2_] d(S–S) equals 3.048 (3) Å, the torsion angle is 102.50 (3) ° (Veith *et al.*, 2005), and in [Fe_2_S_2_(CO)_6_Ca(thf)_4_] described here d(S–S) = 3.129 (3) Å, and the S1–Fe1–Fe2–S2 torsion angle is 107.78 (2) °. The large variability, especially of the S–Fe–Fe–S torsion angle, indicates a pronounced geometric flexibility of the [Fe_2_(CO)_6_(µ-S_2_)] core.

### Experimental

All manipulations were carried out under strictly anaerobic and anhydrous conditions. For the synthesis an ampoule possessing two sections (1 and 2) orientated at right angles to each other was used. In an argon glove box [Fe_2_(CO)_6_(µ-S)_2_] (100 mg, 0.29 mmol), [Ca(thf)_4_(dpp-BIAN)] (241 mg, 0.29 mmol) and a Teflon-coated magnetic stirring bar were placed into section 1. The ampoule was connected to a vacuum condensation line and THF (30 ml) was condensed into the same section cooled by liquid nitrogen. Then, the ampoule was flame sealed and placed on a magnetic stirrer. The reaction mixture was allowed to warm up to room temperature at permanent stirring, which was subsequently continued for 24 h. A black microcrystalline precipitate of crude [Fe_2_(CO)_6_(µ_3_-S)_2_Ca(thf)_4_] was separated by decantation of the solution to section 2 and washed out by THF in the following manner: the ampoule was mounted so that the section 2 was oriented vertically, section 1 containing the black precipitate was located on top horizontally, a recirculation of THF (evaporation into section 2, condensation into section 1 and flowing down to section 2) was achieved by means of a temperature difference between the sections: 1 - 288 K, 2 - 298 K. In a period of a few days this led to enlargement of the crystallites of [Fe_2_(CO)_6_(µ_3_-S)_2_Ca(thf)_4_] and complete removal of dpp-BIAN the solid. After that all volatiles were removed from the section 1 by cooling of section 2 with liquid nitrogen. Section 1 with black crystalline [Fe_2_(CO)_6_(µ_3_-S)_2_Ca(thf)_4_] (yield 60%) was flame sealed and opened in a glovebox. The compound is extremely air sensitive, so satisfactory analytical data were not obtained. Uniformity of the sample was proved by IR spectroscopy which exhibits the expected characteristic pattern in the CO region (cm^-1^): 2083 m, 2042 s, 2024 s, 2006 s, 1976 s, 1943 s, 1904 s (in mineral oil). Single crystals suitable for X-ray analysis were found in the black crystalline mass. Selection of the crystal for X-ray analysis was performed under a microscope in a glovebox. The crystal was taken up in a drop of mineral oil and was immediately mounted on the diffractometer.

### Crystal data

**Table:**

[CaFe_2_S_2_(C_4_H_8_O)_4_(CO)_6_]	*F*(000) = 1392
*M**_r_* = 672.38	*D*_x_ = 1.535 Mg m^−^^3^
Orthorhombic, *P*2_1_2_1_2_1_	Mo *K*α radiation, λ = 0.71073 Å
Hall symbol: P 2ac 2ab	Cell parameters from 7648 reflections
*a* = 10.9189 (4) Å	θ = 2.5–26.2°
*b* = 12.4167 (5) Å	µ = 1.37 mm^−^^1^
*c* = 21.4545 (9) Å	*T* = 150 K
*V* = 2908.7 (2) Å^3^	Prism, black
*Z* = 4	0.25 × 0.11 × 0.08 mm

### Data collection

**Table:**

Bruker–Nonius X8 APEX CCD area-detector diffractometer	5951 independent reflections
Radiation source: fine-focus sealed tube	5319 reflections with *I* > 2σ(*I*)
Graphite monochromator	*R*_int_ = 0.033
Detector resolution: 25 pixels mm^-1^	θ_max_ = 26.4°, θ_min_ = 1.9°
φ scans	*h* = −13→11
Absorption correction: multi-scan (*SADABS*; Bruker, 2004)	*k* = −13→15
*T*_min_ = 0.726, *T*_max_ = 0.899	*l* = −26→26
21313 measured reflections	

### Refinement

**Table:**

Refinement on *F*^2^	Secondary atom site location: difference Fourier map
Least-squares matrix: full	Hydrogen site location: inferred from neighbouring sites
*R*[*F*^2^ > 2σ(*F*^2^)] = 0.027	H-atom parameters constrained
*wR*(*F*^2^) = 0.057	*w* = 1/[σ^2^(*F*_o_^2^) + (0.029*P*)^2^] where *P* = (*F*_o_^2^ + 2*F*_c_^2^)/3
*S* = 1.01	(Δ/σ)_max_ = 0.002
5951 reflections	Δρ_max_ = 0.36 e Å^−^^3^
334 parameters	Δρ_min_ = −0.32 e Å^−^^3^
0 restraints	Absolute structure: Flack (1983), 2601 Friedel pairs
Primary atom site location: structure-invariant direct methods	Flack parameter: 0.006 (11)

### Special details

**Table:**

Geometry. All e.s.d.'s (except the e.s.d. in the dihedral angle between two l.s. planes) are estimated using the full covariance matrix. The cell e.s.d.'s are taken into account individually in the estimation of e.s.d.'s in distances, angles and torsion angles; correlations between e.s.d.'s in cell parameters are only used when they are defined by crystal symmetry. An approximate (isotropic) treatment of cell e.s.d.'s is used for estimating e.s.d.'s involving l.s. planes.
Refinement. Refinement of *F*^2^ against ALL reflections. The weighted *R*-factor *wR* and goodness of fit *S* are based on *F*^2^, conventional *R*-factors *R* are based on *F*, with *F* set to zero for negative *F*^2^. The threshold expression of *F*^2^ > σ(*F*^2^) is used only for calculating *R*-factors(gt) *etc*. and is not relevant to the choice of reflections for refinement. *R*-factors based on *F*^2^ are statistically about twice as large as those based on *F*, and *R*- factors based on ALL data will be even larger.

### Fractional atomic coordinates and isotropic or equivalent isotropic displacement parameters (Å^2^)

**Table:**

	*x*	*y*	*z*	*U*_iso_*/*U*_eq_	
Fe1	0.79820 (3)	0.40586 (3)	0.674330 (16)	0.02253 (8)	
Fe2	0.82499 (3)	0.59531 (3)	0.635135 (17)	0.02489 (9)	
Ca1	0.51290 (4)	0.49436 (4)	0.60998 (2)	0.01931 (10)	
S1	0.67335 (5)	0.54573 (5)	0.70434 (3)	0.02536 (14)	
S2	0.75029 (6)	0.45765 (5)	0.57341 (3)	0.02487 (14)	
O1	1.02452 (18)	0.32234 (16)	0.61922 (10)	0.0441 (5)	
O2	0.88333 (18)	0.40811 (19)	0.80361 (9)	0.0479 (5)	
O3	0.64995 (17)	0.21109 (14)	0.69010 (9)	0.0348 (5)	
O4	1.04619 (17)	0.58348 (18)	0.55847 (9)	0.0467 (5)	
O5	0.9470 (2)	0.68878 (18)	0.74443 (11)	0.0561 (6)	
O6	0.71495 (19)	0.78658 (16)	0.57777 (10)	0.0459 (5)	
O11	0.35797 (14)	0.48969 (14)	0.68605 (8)	0.0259 (4)	
O21	0.44972 (16)	0.30895 (12)	0.59998 (8)	0.0287 (4)	
O31	0.43354 (16)	0.49442 (14)	0.50679 (8)	0.0284 (4)	
O41	0.44134 (17)	0.67554 (12)	0.60327 (8)	0.0278 (4)	
C1	0.9361 (3)	0.3532 (2)	0.64150 (13)	0.0301 (6)	
C2	0.8518 (2)	0.4082 (2)	0.75288 (12)	0.0302 (6)	
C3	0.7062 (2)	0.2881 (2)	0.68292 (11)	0.0254 (5)	
C4	0.9604 (2)	0.5880 (2)	0.58892 (13)	0.0325 (6)	
C5	0.8992 (3)	0.6536 (2)	0.70162 (15)	0.0353 (7)	
C6	0.7565 (2)	0.7112 (2)	0.60022 (13)	0.0317 (6)	
C12	0.3812 (3)	0.4509 (2)	0.74846 (13)	0.0350 (7)	
H12A	0.4171	0.5084	0.7747	0.042*	
H12B	0.4378	0.3887	0.7478	0.042*	
C13	0.2579 (3)	0.4183 (3)	0.77249 (15)	0.0525 (9)	
H13A	0.2547	0.4221	0.8186	0.063*	
H13B	0.2365	0.3444	0.7590	0.063*	
C14	0.1737 (3)	0.5006 (3)	0.74314 (15)	0.0529 (9)	
H14A	0.0893	0.4724	0.7394	0.063*	
H14B	0.1722	0.5681	0.7677	0.063*	
C15	0.2301 (2)	0.5187 (2)	0.67990 (14)	0.0345 (6)	
H15A	0.1895	0.4731	0.6482	0.041*	
H15B	0.2218	0.5951	0.6673	0.041*	
C22	0.3577 (3)	0.2470 (2)	0.63353 (15)	0.0365 (7)	
H22A	0.2800	0.2878	0.6367	0.044*	
H22B	0.3865	0.2293	0.6761	0.044*	
C23	0.3400 (3)	0.1453 (2)	0.59526 (15)	0.0431 (8)	
H23A	0.3138	0.0842	0.6218	0.052*	
H23B	0.2790	0.1563	0.5617	0.052*	
C24	0.4666 (3)	0.1266 (2)	0.56874 (14)	0.0385 (7)	
H24A	0.4635	0.0811	0.5309	0.046*	
H24B	0.5214	0.0926	0.5998	0.046*	
C25	0.5076 (3)	0.2404 (2)	0.55347 (13)	0.0347 (7)	
H25A	0.5979	0.2463	0.5559	0.042*	
H25B	0.4811	0.2609	0.5110	0.042*	
C32	0.3135 (2)	0.4568 (2)	0.48866 (12)	0.0314 (6)	
H32A	0.2548	0.5175	0.4869	0.038*	
H32B	0.2828	0.4027	0.5187	0.038*	
C33	0.3296 (3)	0.4070 (3)	0.42478 (13)	0.0442 (7)	
H33A	0.3264	0.3275	0.4272	0.053*	
H33B	0.2651	0.4323	0.3958	0.053*	
C34	0.4545 (4)	0.4447 (4)	0.40367 (16)	0.0787 (14)	
H34A	0.5097	0.3822	0.3978	0.094*	
H34B	0.4478	0.4837	0.3636	0.094*	
C35	0.5017 (3)	0.5140 (3)	0.45056 (13)	0.0566 (10)	
H35A	0.4928	0.5902	0.4377	0.068*	
H35B	0.5898	0.4991	0.4575	0.068*	
C42	0.3891 (3)	0.7332 (2)	0.55093 (13)	0.0458 (9)	
H42A	0.3244	0.6896	0.5307	0.055*	
H42B	0.4530	0.7500	0.5197	0.055*	
C43	0.3355 (3)	0.8361 (2)	0.57789 (14)	0.0410 (7)	
H43A	0.3371	0.8954	0.5471	0.049*	
H43B	0.2504	0.8250	0.5924	0.049*	
C44	0.4212 (3)	0.8579 (2)	0.63176 (13)	0.0336 (6)	
H44A	0.3818	0.9041	0.6636	0.040*	
H44B	0.4977	0.8927	0.6174	0.040*	
C45	0.4458 (3)	0.7462 (2)	0.65700 (12)	0.0323 (7)	
H45A	0.5272	0.7430	0.6772	0.039*	
H45B	0.3826	0.7256	0.6879	0.039*	

### Atomic displacement parameters (Å^2^)

**Table:**

	*U*^11^	*U*^22^	*U*^33^	*U*^12^	*U*^13^	*U*^23^
Fe1	0.02134 (17)	0.02364 (17)	0.02262 (17)	−0.00130 (15)	−0.00090 (15)	−0.00073 (16)
Fe2	0.02297 (18)	0.02352 (18)	0.02818 (19)	−0.00199 (15)	0.00003 (16)	−0.00004 (16)
Ca1	0.0206 (2)	0.0174 (2)	0.0200 (2)	−0.00045 (19)	−0.0008 (2)	−0.0010 (2)
S1	0.0212 (3)	0.0320 (3)	0.0229 (3)	0.0001 (3)	−0.0003 (3)	−0.0069 (3)
S2	0.0221 (3)	0.0320 (3)	0.0205 (3)	0.0010 (3)	0.0008 (3)	−0.0038 (3)
O1	0.0291 (11)	0.0478 (12)	0.0555 (14)	0.0062 (9)	0.0066 (10)	−0.0083 (11)
O2	0.0443 (12)	0.0709 (14)	0.0286 (11)	−0.0113 (11)	−0.0107 (9)	0.0019 (11)
O3	0.0381 (11)	0.0308 (10)	0.0355 (11)	−0.0085 (9)	0.0007 (9)	0.0050 (9)
O4	0.0287 (11)	0.0664 (15)	0.0450 (13)	0.0017 (10)	0.0092 (10)	0.0121 (11)
O5	0.0552 (15)	0.0513 (14)	0.0618 (15)	−0.0080 (12)	−0.0199 (13)	−0.0194 (12)
O6	0.0474 (13)	0.0367 (12)	0.0536 (13)	0.0089 (10)	0.0131 (11)	0.0177 (11)
O11	0.0215 (9)	0.0311 (9)	0.0251 (9)	0.0010 (7)	0.0008 (7)	0.0064 (8)
O21	0.0337 (10)	0.0173 (8)	0.0351 (11)	−0.0034 (8)	0.0009 (8)	−0.0027 (8)
O31	0.0287 (9)	0.0347 (9)	0.0216 (9)	−0.0068 (8)	−0.0037 (7)	−0.0046 (8)
O41	0.0457 (11)	0.0165 (8)	0.0211 (9)	0.0027 (8)	−0.0039 (8)	−0.0005 (7)
C1	0.0288 (15)	0.0287 (14)	0.0327 (15)	−0.0034 (11)	−0.0052 (13)	0.0009 (12)
C2	0.0258 (14)	0.0332 (14)	0.0317 (15)	−0.0054 (12)	0.0007 (11)	0.0002 (13)
C3	0.0259 (13)	0.0301 (14)	0.0201 (13)	0.0042 (11)	−0.0003 (11)	0.0015 (11)
C4	0.0297 (15)	0.0330 (15)	0.0348 (16)	−0.0065 (13)	−0.0056 (13)	0.0094 (13)
C5	0.0298 (15)	0.0282 (14)	0.0481 (18)	−0.0011 (12)	−0.0051 (14)	−0.0031 (14)
C6	0.0301 (14)	0.0309 (15)	0.0343 (16)	−0.0048 (12)	0.0097 (13)	0.0020 (13)
C12	0.0436 (17)	0.0327 (15)	0.0286 (15)	0.0051 (13)	−0.0018 (12)	0.0076 (12)
C13	0.070 (2)	0.0479 (19)	0.0393 (18)	−0.0196 (18)	0.0223 (17)	0.0036 (15)
C14	0.0278 (16)	0.074 (2)	0.057 (2)	−0.0113 (17)	0.0132 (15)	−0.0199 (18)
C15	0.0214 (14)	0.0376 (16)	0.0444 (16)	−0.0007 (11)	−0.0016 (12)	0.0003 (14)
C22	0.0353 (16)	0.0262 (14)	0.0481 (18)	−0.0054 (11)	0.0045 (14)	−0.0005 (14)
C23	0.0445 (18)	0.0223 (14)	0.062 (2)	−0.0072 (13)	−0.0101 (16)	−0.0049 (14)
C24	0.0493 (19)	0.0211 (14)	0.0452 (18)	0.0025 (12)	−0.0067 (15)	−0.0104 (12)
C25	0.0444 (18)	0.0262 (14)	0.0336 (16)	0.0033 (12)	−0.0011 (14)	−0.0063 (12)
C32	0.0251 (13)	0.0353 (14)	0.0337 (14)	0.0005 (12)	−0.0032 (12)	0.0006 (12)
C33	0.0467 (18)	0.0561 (19)	0.0298 (15)	−0.0167 (17)	−0.0119 (14)	−0.0038 (15)
C34	0.083 (3)	0.117 (3)	0.037 (2)	−0.055 (3)	0.0211 (19)	−0.026 (2)
C35	0.0482 (19)	0.093 (3)	0.0284 (16)	−0.0292 (19)	0.0058 (14)	−0.0035 (17)
C42	0.085 (3)	0.0277 (16)	0.0253 (16)	0.0141 (15)	−0.0124 (16)	0.0010 (12)
C43	0.056 (2)	0.0279 (15)	0.0388 (17)	0.0138 (14)	−0.0080 (15)	0.0006 (13)
C44	0.0411 (16)	0.0207 (13)	0.0388 (16)	0.0087 (11)	−0.0040 (14)	−0.0054 (12)
C45	0.0480 (18)	0.0257 (14)	0.0232 (15)	0.0006 (12)	−0.0011 (12)	−0.0066 (11)

### Geometric parameters (Å, º)

**Table:**

Fe1—C3	1.784 (3)	C14—C15	1.507 (4)
Fe1—C2	1.784 (3)	C14—H14A	0.9900
Fe1—C1	1.786 (3)	C14—H14B	0.9900
Fe1—Fe2	2.5152 (5)	C15—H15A	0.9900
Fe1—S1	2.2999 (7)	C15—H15B	0.9900
Fe1—S2	2.3185 (7)	C22—C23	1.519 (4)
Fe2—S1	2.3077 (7)	C22—H22A	0.9900
Fe2—S2	2.3110 (7)	C22—H22B	0.9900
Ca1—S1	2.7523 (8)	C23—C24	1.513 (4)
Ca1—S2	2.7463 (8)	C23—H23A	0.9900
Ca1—Fe1	3.5802 (6)	C23—H23B	0.9900
Ca1—Fe2	3.6708 (6)	C24—C25	1.517 (4)
Fe2—C4	1.783 (3)	C24—H24A	0.9900
Fe2—C6	1.787 (3)	C24—H24B	0.9900
Fe2—C5	1.793 (3)	C25—H25A	0.9900
Ca1—O11	2.3513 (17)	C25—H25B	0.9900
Ca1—O31	2.3773 (17)	C32—C33	1.514 (4)
Ca1—O41	2.3858 (16)	C32—H32A	0.9900
Ca1—O21	2.4128 (16)	C32—H32B	0.9900
O1—C1	1.144 (3)	C33—C34	1.511 (4)
O2—C2	1.142 (3)	C33—H33A	0.9900
O3—C3	1.147 (3)	C33—H33B	0.9900
O4—C4	1.143 (3)	C34—C35	1.421 (4)
O5—C5	1.143 (3)	C34—H34A	0.9900
O6—C6	1.146 (3)	C34—H34B	0.9900
O11—C12	1.445 (3)	C35—H35A	0.9900
O11—C15	1.448 (3)	C35—H35B	0.9900
O21—C22	1.456 (3)	C42—C43	1.519 (4)
O21—C25	1.456 (3)	C42—H42A	0.9900
O31—C35	1.438 (3)	C42—H42B	0.9900
O31—C32	1.445 (3)	C43—C44	1.511 (4)
O41—C42	1.449 (3)	C43—H43A	0.9900
O41—C45	1.450 (3)	C43—H43B	0.9900
C12—C13	1.498 (4)	C44—C45	1.513 (3)
C12—H12A	0.9900	C44—H44A	0.9900
C12—H12B	0.9900	C44—H44B	0.9900
C13—C14	1.512 (5)	C45—H45A	0.9900
C13—H13A	0.9900	C45—H45B	0.9900
C13—H13B	0.9900		
			
C3—Fe1—C2	95.79 (12)	O11—C12—H12A	110.8
C3—Fe1—C1	102.44 (11)	C13—C12—H12A	110.8
C2—Fe1—C1	95.85 (12)	O11—C12—H12B	110.8
C3—Fe1—S1	104.86 (8)	C13—C12—H12B	110.8
C2—Fe1—S1	85.27 (9)	H12A—C12—H12B	108.9
C1—Fe1—S1	152.42 (9)	C12—C13—C14	102.7 (2)
C3—Fe1—S2	101.35 (8)	C12—C13—H13A	111.2
C2—Fe1—S2	162.08 (9)	C14—C13—H13A	111.2
C1—Fe1—S2	85.60 (9)	C12—C13—H13B	111.2
S1—Fe1—S2	85.31 (2)	C14—C13—H13B	111.2
C3—Fe1—Fe2	150.09 (8)	H13A—C13—H13B	109.1
C2—Fe1—Fe2	105.19 (9)	C15—C14—C13	103.2 (2)
C1—Fe1—Fe2	96.45 (9)	C15—C14—H14A	111.1
S1—Fe1—Fe2	57.06 (2)	C13—C14—H14A	111.1
S2—Fe1—Fe2	56.95 (2)	C15—C14—H14B	111.1
C3—Fe1—Ca1	78.55 (8)	C13—C14—H14B	111.1
C2—Fe1—Ca1	130.14 (9)	H14A—C14—H14B	109.1
C1—Fe1—Ca1	133.92 (9)	O11—C15—C14	105.9 (2)
S1—Fe1—Ca1	50.242 (18)	O11—C15—H15A	110.6
S2—Fe1—Ca1	50.091 (18)	C14—C15—H15A	110.6
Fe2—Fe1—Ca1	71.653 (13)	O11—C15—H15B	110.6
C4—Fe2—C6	98.93 (12)	C14—C15—H15B	110.6
C4—Fe2—C5	95.07 (13)	H15A—C15—H15B	108.7
C6—Fe2—C5	101.39 (12)	O21—C22—C23	105.1 (2)
C4—Fe2—S1	159.96 (9)	O21—C22—H22A	110.7
C6—Fe2—S1	100.61 (9)	C23—C22—H22A	110.7
C5—Fe2—S1	85.40 (10)	O21—C22—H22B	110.7
C4—Fe2—S2	86.35 (9)	C23—C22—H22B	110.7
C6—Fe2—S2	101.99 (9)	H22A—C22—H22B	108.8
C5—Fe2—S2	156.06 (9)	C24—C23—C22	102.4 (2)
S1—Fe2—S2	85.31 (2)	C24—C23—H23A	111.3
C4—Fe2—Fe1	103.58 (9)	C22—C23—H23A	111.3
C6—Fe2—Fe1	147.66 (9)	C24—C23—H23B	111.3
C5—Fe2—Fe1	99.45 (9)	C22—C23—H23B	111.3
S1—Fe2—Fe1	56.767 (19)	H23A—C23—H23B	109.2
S2—Fe2—Fe1	57.24 (2)	C23—C24—C25	102.0 (2)
C4—Fe2—Ca1	132.13 (9)	C23—C24—H24A	111.4
C6—Fe2—Ca1	79.91 (9)	C25—C24—H24A	111.4
C5—Fe2—Ca1	132.39 (10)	C23—C24—H24B	111.4
S1—Fe2—Ca1	48.504 (18)	C25—C24—H24B	111.4
S2—Fe2—Ca1	48.359 (18)	H24A—C24—H24B	109.2
Fe1—Fe2—Ca1	67.779 (13)	O21—C25—C24	105.6 (2)
O11—Ca1—O31	112.59 (6)	O21—C25—H25A	110.6
O11—Ca1—O41	80.18 (6)	C24—C25—H25A	110.6
O31—Ca1—O41	79.87 (6)	O21—C25—H25B	110.6
O11—Ca1—O21	80.36 (6)	C24—C25—H25B	110.6
O31—Ca1—O21	79.24 (6)	H25A—C25—H25B	108.8
O41—Ca1—O21	143.19 (6)	O31—C32—C33	105.7 (2)
O11—Ca1—S2	150.84 (5)	O31—C32—H32A	110.6
O31—Ca1—S2	94.48 (5)	C33—C32—H32A	110.6
O41—Ca1—S2	116.64 (5)	O31—C32—H32B	110.6
O21—Ca1—S2	94.94 (5)	C33—C32—H32B	110.6
O11—Ca1—S1	87.31 (5)	H32A—C32—H32B	108.7
O31—Ca1—S1	156.48 (5)	C34—C33—C32	104.5 (2)
O41—Ca1—S1	91.96 (5)	C34—C33—H33A	110.9
O21—Ca1—S1	117.94 (5)	C32—C33—H33A	110.9
S2—Ca1—S1	69.38 (2)	C34—C33—H33B	110.9
O11—Ca1—Fe1	110.53 (4)	C32—C33—H33B	110.9
O31—Ca1—Fe1	132.56 (5)	H33A—C33—H33B	108.9
O41—Ca1—Fe1	126.70 (5)	C35—C34—C33	107.6 (3)
O21—Ca1—Fe1	89.44 (4)	C35—C34—H34A	110.2
S2—Ca1—Fe1	40.358 (15)	C33—C34—H34A	110.2
S1—Ca1—Fe1	39.970 (15)	C35—C34—H34B	110.2
O11—Ca1—Fe2	125.05 (5)	C33—C34—H34B	110.2
O31—Ca1—Fe2	118.37 (4)	H34A—C34—H34B	108.5
O41—Ca1—Fe2	89.48 (5)	C34—C35—O31	107.7 (3)
O21—Ca1—Fe2	127.18 (4)	C34—C35—H35A	110.2
S2—Ca1—Fe2	38.966 (16)	O31—C35—H35A	110.2
S1—Ca1—Fe2	38.903 (15)	C34—C35—H35B	110.2
Fe1—Ca1—Fe2	40.568 (10)	O31—C35—H35B	110.2
Fe1—S1—Fe2	66.17 (2)	H35A—C35—H35B	108.5
Fe1—S1—Ca1	89.79 (2)	O41—C42—C43	105.8 (2)
Fe2—S1—Ca1	92.59 (2)	O41—C42—H42A	110.6
Fe2—S2—Fe1	65.82 (2)	C43—C42—H42A	110.6
Fe2—S2—Ca1	92.67 (2)	O41—C42—H42B	110.6
Fe1—S2—Ca1	89.55 (2)	C43—C42—H42B	110.6
C12—O11—C15	109.66 (19)	H42A—C42—H42B	108.7
C12—O11—Ca1	121.67 (15)	C44—C43—C42	101.7 (2)
C15—O11—Ca1	128.63 (16)	C44—C43—H43A	111.4
C22—O21—C25	109.24 (18)	C42—C43—H43A	111.4
C22—O21—Ca1	131.11 (15)	C44—C43—H43B	111.4
C25—O21—Ca1	119.65 (15)	C42—C43—H43B	111.4
C35—O31—C32	107.36 (19)	H43A—C43—H43B	109.3
C35—O31—Ca1	126.33 (16)	C43—C44—C45	102.7 (2)
C32—O31—Ca1	125.54 (14)	C43—C44—H44A	111.2
C42—O41—C45	109.29 (18)	C45—C44—H44A	111.2
C42—O41—Ca1	129.91 (15)	C43—C44—H44B	111.2
C45—O41—Ca1	120.79 (14)	C45—C44—H44B	111.2
O1—C1—Fe1	177.8 (3)	H44A—C44—H44B	109.1
O2—C2—Fe1	178.1 (2)	O41—C45—C44	105.3 (2)
O3—C3—Fe1	177.5 (2)	O41—C45—H45A	110.7
O4—C4—Fe2	178.9 (2)	C44—C45—H45A	110.7
O5—C5—Fe2	178.7 (3)	O41—C45—H45B	110.7
O6—C6—Fe2	178.5 (2)	C44—C45—H45B	110.7
O11—C12—C13	104.6 (2)	H45A—C45—H45B	108.8

### Hydrogen-bond geometry (Å, º)

**Table:**

*D*—H···*A*	*D*—H	H···*A*	*D*···*A*	*D*—H···*A*
C34—H34*A*···O1^i^	0.99	2.57	3.437 (5)	146
C34—H34*B*···O2^ii^	0.99	2.62	3.329 (4)	129
C42—H42*A*···O6^iii^	0.99	2.63	3.362 (4)	131
C45—H45*B*···O3^iv^	0.99	2.65	3.470 (3)	141
C15—H15*A*···O1^v^	0.99	2.67	3.561 (3)	150

Symmetry codes: (i) *x*−1/2, −*y*+1/2, −*z*+1; (ii) −*x*+3/2, −*y*+1, *z*−1/2; (iii) *x*−1/2, −*y*+3/2, −*z*+1; (iv) −*x*+1, *y*+1/2, −*z*+3/2; (v) *x*−1, *y*, *z*.
